# Comprehensive Analysis of the Relationship Between Leisure Constraints Negotiation and Leisure Participation Within the Korean Context

**DOI:** 10.3389/fpsyg.2022.733200

**Published:** 2022-03-24

**Authors:** Eui Jae Kim, Seong Man Park, Hyun Wook Kang

**Affiliations:** Dankook University, Yongin, South Korea

**Keywords:** leisure constraints negotiation, leisure participation, leisure activities, meta-analysis, Korea

## Abstract

The purpose of this study was to identify relationship between leisure constraint negotiations and leisure activity participation through meta-analysis within the Korean context. Through this study, the inconsistent research results of previous studies are explained by comprehensively clarifying the relationship between the two variables and identifying a third variable that controls the relationship. The efforts of this project are expected to provide useful data that can be used for future research and to seek ways of increasing participation in leisure activities. In order to achieve the purpose of this study, the research questions set in this study are as follows. First, what is the magnitude of the effect of the correlation coefficient between leisure constraint negotiations and leisure participation? Second, what are the variables that control the relationship between leisure constraint negotiations and leisure participation? Materials were collected by utilizing Research Information Sharing Service, DBpia, Korean studies Information Service System, and Korea Citation Index. Finally, 20 pieces of research materials were selected with a total of 6,843 participants. The conclusions drawn from the research questions set in this study are as follows. First, leisure constraint negotiations and leisure participation are in a static relationship, and their level is considerable. In other words, efforts to overcome leisure constraints increase participation in leisure activities. Second, gender, age, and type of active leisure activities are major variables that control the relationship between leisure constraint negotiations and leisure participation. Finally, the limitation of this study and future research orientation were discussed.

## Introduction

Today, leisure activities are important to individuals in many ways. Healthy leisure activities increase subjective happiness (Choi et al., 2019), affect successful aging (Lee and Ahn, 2010), and are associated with low mortality rates (Arrieta and Russell, 2008). In addition, studies have reported a use of leisure time is believed to reduce the level of Internet addiction (Lin et al., 2009), positively affects productivity in the workplace (Corgnet et al., 2015), and improves physical and mental health (Iwasaki et al., 2001). In addition, individuals’ well-being and stress are closely related with their leisure activities, and the lack of leisure activities can bring negative consequences to their stresses and well-being (Christopher et al., 2018).

It should also be noted that many empirical studies have been conducted in academia to identify the factors that affect participation in leisure activities. Amongst these factors, the relationship between participation in leisure activities was observed in terms of leisure constraints, leisure motivation, self-efficacy, leisure attitude, subjective norms, perceived behavioral control, aspiration, and leisure constraint negotiation variables (Alexandris et al., 2007; Son et al., 2008; Kim, 2009; Lee and Scott, 2009; Choi et al., 2011; Hung and Petrick, 2012; Ryu and Lee, 2012; Ma and Ma, 2014; Son and Kim, 2014; Moghimehfar et al., 2018; Lee, 2019; Lim and Lee, 2019). In particular, leisure constraint negotiations have consistently been reported to be one of the major determinants of participation in leisure activities.

Leisure constraint negotiations are defined as an individual’s willingness and efforts to reduce the impact of constraints limiting leisure participation or promote leisure participation (Jackson et al., 1993; Mannell and Kleiber, 1997). Research on leisure constraint negotiations began to come to life with Crawford et al. (1991) and Jackson and Rucks (1995). Specifically, Crawford et al. (1991) emphasized the importance of the leisure constraint negotiation variable that mediates the relationship between leisure constraints and participation in leisure activities. Additionally, their research found that most of the constraints could be overcome through the negotiation process. Following these seminal works, studies related to leisure constraint negotiations have continued to be conducted by many researchers. For example, Hubbard and Mannell (2001) also observed that leisure constraint factors negatively affect participation in leisure activities through the constraint-effects-mitigation model but can be mitigated through the negotiation process.

Meanwhile, studies on the relationship between leisure constraint negotiations and participation in leisure have been continuously conducted, and in many studies, significant positive correlations have been observed between the two variables (Kim, 2008, 2009, 2018; Choi, 2009; Lee and Scott, 2009; Choi et al., 2011; Choi and Han, 2012; Kocak, 2017; Kim et al., 2019). However, it should also be noted that some studies have either found no significant relationship between the two variables (Kim and Yoon, 2012; Son and Kim, 2014), or the results showed a negative relationship between the two variables (Jin, 2010; Han, 2011; Kim et al., 2011). In addition, looking at the correlation coefficient between the two variables in the above mentioned studies, it was observed to be between −0.20 and 0.77, indicating that the direction and level of the relationship between leisure constraint negotiations and leisure participation are not consistent. This result suggests that there is a need to comprehensively examine the relationship between the two variables.

Another key aspect of this project is the use of meta-analysis, which is an analysis of analyses (Glass, 1976) to systemically review research results (Borenstein et al., 2009). In other words, this method is a comprehensive analysis method that systematically and quantitatively analyzes various research results on the same subject (Hwang, 2014) while providing an objective basis for generalizing research results. Furthermore, for individual researchers, there are practical difficulties in conducting large-sample studies. Therefore, if the available resources of social scientists are considered, a meta-analysis that integrates research results derived from small samples can be a good solution (Hunter and Schmidt, 2004).

Meanwhile, a meta-analysis was attempted in a study by Han et al. (2018) for the first time to comprehensively analyze the effectiveness of leisure constraint negotiations. However, it is somewhat difficult to specifically explain the relationship between leisure constraint negotiations and participation in leisure due to a large number of variables (leisure satisfaction, acceptance intention, recreational specialization, serious leisure, participation intention, etc.). Yet, the recent work of Blinded (2020) estimated the average effect size between leisure constraints and leisure participation through meta-analysis while also trying to explain the variables that control their relationship. These attempts established the primary basis and need for more scientific studies analyzing the relationship between leisure constraints, leisure constraint negotiations, and leisure participation. Therefore, a comprehensive analysis of the relationship between leisure constraint negotiations and leisure participation would be meaningful work.

With regard to the research on the relationship between leisure constraint negotiations and leisure participation within the Korean context in particular, Han (2011) strongly claims that it would not be easy for Korean adolescents to have time to experience leisure life since they are tied to education during their school years in preparation for university entrance exams. In the case of Korea, the public interests in the education system related to the university entrance exam focus mainly on the fact that that university entrance exams are evaluated as a big factor in determining life, such as those who graduated from prestigious universities occupying a superior position in society in terms of employment (Shin, 2012). In addition, college students in Korea, who are supposed to enjoy enough leisure activities without stress for the college entrance exam, do not experience leisure life due to the lack of time and financial worries (Han, 2011). Thus, this study aims to investigate the relationship between leisure constraint negotiations and leisure participation specifically within the Korean context where the search for the leisure participation starts after their late twenties at the earliest in general.

Based on the above factors, the aim of this study is to conduct a meta-analysis to draw a comprehensive conclusion on the relationship between leisure constraint negotiations and leisure participation. Through this study, the inconsistent research results of previous studies are explained by comprehensively clarifying the relationship between the two variables and identifying a third variable that controls the relationship. The efforts of this project are expected to provide useful data that can be used for future research and to seek ways of increasing participation in leisure activities. In order to achieve the purpose of this study, the research questions set in this study are as follows. First, what is the magnitude of the effect of the correlation coefficient between leisure constraint negotiations and leisure participation? Second, what are the variables that control the relationship between leisure constraint negotiations and leisure participation?

## Research Methods

The principle employed in the research method of this study is based on journal article reporting standards for quantitative research of the APA publications and communications board task force report (Appelbaum et al., 2018).

### Data Collection and Selection

**FIGURE 1:**
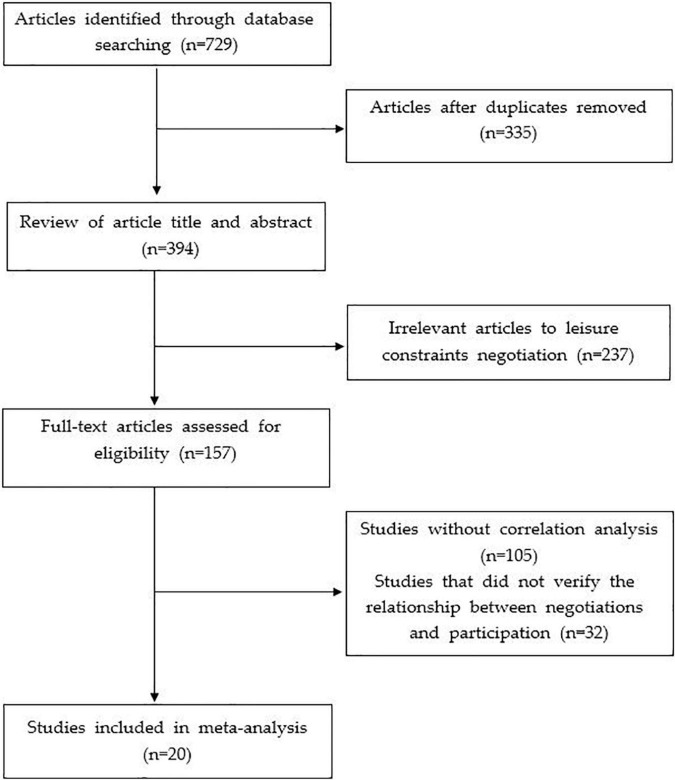
PRISMA flow chart.

For the meta-analysis of the relationship between leisure constraint negotiations and participation in leisure activities, data was collected from the theses and academic journal articles published in Korea for the past 20 years (2000∼2019). The reason to limit the analysis to the last 20 years within the Korean context is that the research has been actively conducted since the research on leisure constraint negotiations began to appear overseas during the 1990s by Crawford et al. (1991); Jackson and Rucks (1995), and Mannell and Kleiber (1997). Online DB were used, such as Korea Academic Information Service (2020), the Korea Research Foundation (2020), the Academic Research Information Service (2020), and Nuri Media DB-PIA (2020) for the collection of data to be analyzed. The main keywords were “Leisure Constraint Negotiation” and “Leisure Constraint Overcome” in Korean. A total of 729 studies were primarily searched through the online DB, and only studies that satisfy the following criteria were chosen for the analysis target. First, the relationship between leisure constraint negotiations and participation in leisure was verified, and second, studies that presented the results of the correlation analysis between the two variables were selected. Therefore, studies that did not verify the relationship between leisure constraint negotiations and leisure participation, and studies that verified the relationship between the two variables but did not present the results of the correlation analysis, were excluded from the analysis target selection process. On the other hand, in the case of a duplicate study in which the thesis and the journal consisted of the same data, the dissertation was selected as an analysis target, excluding the academic journals that simply summarized the thesis. Through this process, a total of 20 studies (i.e., 11 articles and nine dissertations) were selected as targets for meta-analysis. The detailed data collection and selection process is presented in Figure 1, and the list of the selected research materials is presented in Table 1.

**TABLE 1:** Lists of analyzed papers for meta-analysis.

References	Publication type	Sample size
Choi et al. (2011)	Publication	206
Kim (2008)	Dissertation	164
Han (2011)	Publication	382
Son and Kim (2014)	Publication	337
Jin (2010)	Dissertation	718
Kim (2009)	Publication	298
Kim and Yoon (2012)	Publication	210
Lim (2018)	Dissertation	559
Kim et al. (2019)	Publication	212
Ko et al. (2019)	Publication	490
Ko (2019)	Dissertation	497
Choi (2009)	Publication	323
Kim (2015)	Dissertation	389
Kim (2018)	Dissertation	274
Choi and Han (2012)	Publication	371
Koh and Choi (2016)	Publication	189
Do (2019)	Dissertation	276
Kim et al. (2011)	Publication	194
Sung (2010)	Dissertation	463
Lee (2019)	Dissertation	291

### Coding

Information necessary to calculate the effect size from the 20 studies selected as the final analysis target was entered in the coding form which includes ID, author, title of paper, publication year and type, participants’ profile, leisure activity type, leisure constraint negotiation variables, leisure participation variables, sample size, and information of statistics. The coding work was conducted by reflecting the opinions of one professor of leisure and recreation with those of an experienced person performing meta-analysis research, and if there were differences between them, the coding items were modified and supplemented through continuous discussion. Meanwhile, the first (preliminary) coding was conducted by the first author, and then the recoding (secondary) was performed on the final analysis target research, comparing and correcting any inconsistencies with the first coding data. Through this process, the final coding items were configured. In the process of coding, few differences among coders were found, and thus the inter-coder agreements were not separately calculated.

### Data Analysis

#### Effect Size Calculation and Interpretation

For the calculation of the effect size, the formula for calculating the correlation coefficient effect size was used, and it was converted to Fisher’s Z scale for the standardization process (Borenstein et al., 2009). According to the criteria of Cohen (1988), the effect size was interpreted as a small size if it was smaller than 0.1, a medium size if it was about 0.3, and a large size if it was larger than 0.5.

#### Homogeneity Test

Whether the effect sizes extracted from the study to be analyzed could be considered to be extracted from the same population was confirmed by the homogeneity test (Borenstein et al., 2009). In this study, Q statistics were used as a method for verifying the homogeneity. Q statistics were only verified through the null hypothesis, which states that the size of the population effect of the analyzed study was the same and was affected by the number of studies. In addition to the use of Q statistics, I2 statistics were calculated (Hwang, 2014). In general, when the significance probability for Q statistics is less than 0.10 and the I2 statistic is more that 50%, the heterogeneity of the effect size is considered to be significant (Higgins and Green, 2011). As a result of the homogeneity test, the I2 value, which is the ratio of the actual variance between studies to the total variance, was shown high as 99.093, and the Q value was 2095.763, showing a significant difference at the significance level of 0.01, which shows that the effect sizes were found to be heterogeneous. Therefore, in this study, the total effect size was estimated using a random-effects model, and sub-group analysis was performed to identify the cause of heterogeneity.

#### Publication Bias

Publication bias means that studies with positive findings occupy a large proportion of studies subject to meta-analysis, and as a result, meta-analysis results are overestimated (Borenstein et al., 2009). In this study, publication bias was verified to secure the validity of the meta-analysis results. As a verification method, we examined whether the bias exists visually through a funnel plot.

#### Analysis Tool

As a tool for data analysis, a Comprehensive Meta-Analysis (CMA) Version 2.0 program (Biosta, Inc., Englewood, NJ, United States) was employed.

## Results

### Overall Effect Size on the Relationship Between Leisure Constraint Negotiations and Leisure Participation

**FIGURE 2:**
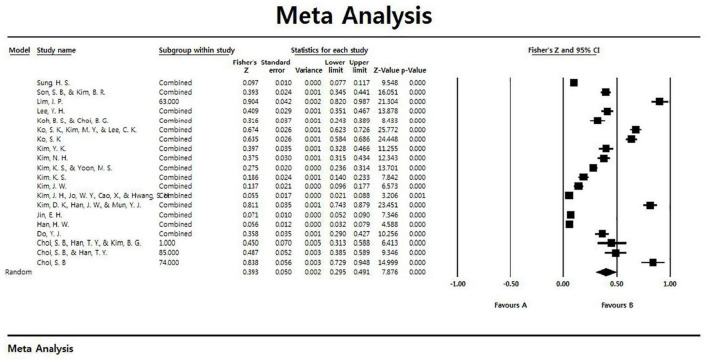
Forest plot.

The results of analyzing the overall effect size on the relationship between leisure constraint negotiations and leisure participation are shown in Table 2. As a result of estimating the total effect size with a random-effects model, the total effect size of the correlation coefficient between leisure constraint negotiation and leisure participation was 0.393, and it was found to be significant in the 95% confidence interval. In addition, a forest plot showing the effect size and average effect size of each study is presented in Figure 2.

**TABLE 2:** Overall effect size.

Model	*n*	ES	95% CI
Random	20	0.393	0.295∼0.491

*n, number studies; ES, effect size; CI, confidence interval.*

### Effect Size of Leisure Constraints Negotiation Sub-Factors and Leisure Participation

The types of leisure constraints specified in this study are intensity adjustment, skill acquisition, companion search, activity cost preparation, time management, energy charging, and aspiration change. The results of analyzing the effect size of sub-factors of leisure constraint negotiations and leisure participation are shown as in Table 3.

**TABLE 3:** Effect size by sub-factor of leisure constraints.

Sub-factor	*n*	ES	−95% CI	+95% CI	*Q*	df	*p*
Intensity adjustment	15	0.203	0.116	0.289	5.311	5	0.379
Skill acquisition	20	0.176	0.101	0.250			
Companion search	21	0.171	0.098	0.244			
Activity cost preparation and Time Management	26	0.219	0.153	0.284			
Energy charging	14	0.113	0.024	0.203			
Aspiration change	15	0.123	0.036	0.209			

*n, number effect sizes; ES, effect size; CI, confidence interval.*

As a result of the analysis, the effect size of each sub-factor of the leisure constraint negotiation was found to be: Leisure activity intensity adjustment 0.203, skill acquisition 0.176, companion search 0.171, activity cost preparation and time management 0.219, energy charging 0.113, and aspiration change 0.123. It was found that the difference between effect sizes was not statistically significant.

### Effect Size of Leisure Constraint Negotiations and Leisure Participation According to Gender

The results of analyzing the effect size of leisure constraint negotiations and leisure participation according to the gender of the study subjects are shown in Table 4. As a result of the analysis, the effect size according to gender was 0.397 for men and 0.160 for women, and the difference between the effect sizes was statistically significant.

**TABLE 4:** Effect size according to gender.

Gender	*n*	ES	−95% CI	+95% CI	*Q*	df	*p*
Male	5	0.397	0.293	0.500	18.146	1	0.000
Female	34	0.160	0.125	0.195			

*n, number effect sizes; ES, effect size; CI, confidence interval.*

### Effect Size of Leisure Constraint Negotiations and Leisure Participation According to Age

The results of analyzing the effect size of leisure constraint negotiations and leisure participation according to the age of the study subjects are shown in Table 5. As a result of the analysis, the effect size according to age was 0.056 in their 20s and 0.360 over the age of 55, and the difference between the effect sizes (*p* = 0.000) was statistically significant (*p* < 0.05).

**TABLE 5:** Effect size according to age.

Age	*n*	ES	−95% CI	+95% CI	*Q*	df	*p*
Over 20	18	0.056	0.002	0.109	35.424	1	0.000
Over 55	9	0.360	0.275	0.444			

*n, number effect sizes; ES, effect size; CI, confidence interval.*

### Effect Size of Leisure Constraints Negotiations and Leisure Participation According to the Types of Leisure Activities

The results of analyzing the effect size of leisure constraint negotiations and leisure participation according to the types of leisure activities are shown in Table 6. As a result of the analysis, the effect size according to the leisure activity type was found to be 0.328 inactive leisure activity and 0.261 active leisure activity, and the difference between effect sizes (*p* = 0.561) was not statistically significant (*p* > 0.05).

**TABLE 6:** Effect size according to the leisure activity type.

Activity type	*n*	ES	−95% CI	+95% CI	*Q*	df	*p*
Inactive	5	0.328	0.109	0.547	0.339	1	0.561
Active	85	0.261	0.209	0.313			

*n, number effect sizes; ES, effect size; CI, confidence interval.*

### Effect Size of Leisure Constraint Negotiations and Leisure Participation According to the Types of Active Leisure Activities

The results of analyzing the effect size of leisure constraint negotiations and leisure participation according to the types of active leisure activities are shown in Table 7. As a result of the analysis, the effect size according to the type of active leisure activity was golf 0.450, dance 0.275, ski/board 0.393, skin scuba diving 0.831, horse riding 0.655, and bicycle 0.375. The difference between the effect sizes was found to be statistically significant.

**TABLE 7:** Effect size according to the types of active leisure activities.

Type of active leisure activities	*n*	ES	−95% CI	+95% CI	*Q*	df	*p*
Golf	1	0.450	0.131	0.769	53.961	5	0.000
Dance	12	0.275	0.183	0.367			
Ski/Board	5	0.393	0.255	0.530			
Skin Scuba Diving	5	0.831	0.691	0.972			
Horse riding	6	0.655	0.532	0.778			
Bicycle	4	0.375	0.219	0.531			

*n, number effect sizes; ES, effect size; CI, confidence interval.*

### Effect Size of Leisure Constraint Negotiations and Leisure Participation According to Publication Type

The results of analyzing the effect size of leisure constraint negotiation and leisure participation according to the type of publication are shown in Table 8. As a result of the analysis, the effect size according to the type of publication was found to be 0.228 in journal articles and 0.206 in thesis papers, and the difference between effect sizes was not statistically significant.

**TABLE 8:** Effect size according to the type of publication.

Publication type	*n*	ES	−95% CI	+95% CI	*Q*	df	*p*
Journal articles	73	0.228	0.175	0.282	0.304	1	0.581
Thesis papers	62	0.206	0.149	0.264			

*n, number effect sizes; ES, effect size; CI, confidence interval.*

### Publication Bias Verification

**FIGURE 3:**
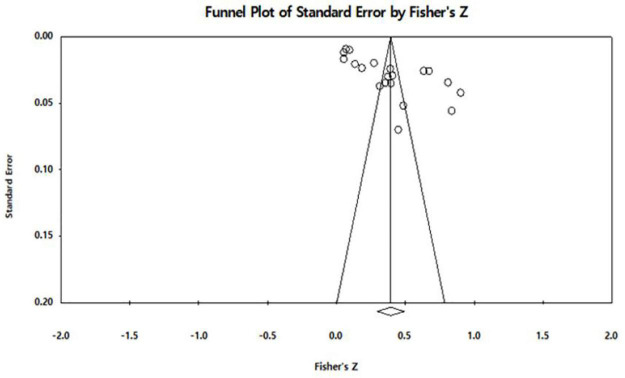
Funnel plot.

To validate the meta-analysis results, a publication bias test was conducted. A funnel plot was employed to determine publication bias. The results of examining the overall distribution through a funnel chart with the x-axis as the effect size (Fisher’s Z) and the y-axis as the standard error, as depicted in Figure 3, confirmed that the effect size was relatively symmetrical around the average.

## Discussion

In this study, a meta-analysis was conducted on 20 studies that verified the relationship between the two variables in order to draw a comprehensive conclusion on the relationship between leisure constraint negotiations and leisure participation. The discussion of the main results of this study is as follows.

First, as a result of analyzing the overall effect size on the relationship between leisure constraint negotiations and leisure participation, a significant positive relationship was observed between the two variables. This result is consistent with the findings and claims from previous studies (Crawford et al., 1991; Jackson and Rucks, 1995; Hubbard and Mannell, 2001; Kocak, 2017) which support the positive inter-relationship between leisure constraint negotiations and leisure participation. Based on this result, it is suggested that leisure constraint negotiation is an important factor for participation in leisure activities, and it is possible to explain that the chances of continuing leisure activities can be increased through the leisure constraint negotiation process. This is somewhat consistent with the meta-analysis review that observed a meaningful static relationship between the dependent variables including, leisure constraint negotiations, leisure satisfaction, recreational specialization, serious leisure, and participation intention (Han et al., 2018). Furthermore, the results of this study are consistent with previous studies, which showed that leisure constraint negotiations have a positive effect on leisure participation (Son et al., 2008; Choi, 2009; Kim, 2009; Lee and Scott, 2009; Ma and Ma, 2014). In a more recent cross sectional study conducted with Japanese and Euro-Canadian adults on the relationships among leisure constraints, leisure constraint negotiation, and leisure enjoyment, Kono and Ito (2021) claim that leisure enjoyment is also positively related with leisure constraint negotiation while enjoyment is negatively related with constraints. On the other hand, the magnitude of the effect on the relationship between leisure constraint negotiations and leisure participation appeared close to the large size (Cohen, 1988). According to a recent meta-analysis study, the magnitude of the effect on the relationship between leisure constraints and leisure participation was observed to be −0.116 (Blinded, 2020). This shows that leisure constraint negotiation is a stronger predictor of participation in leisure activities than leisure constraint. In other words, while it is true that various constraints experienced by participants in leisure activities may stop or decrease participation in leisure activities, the findings of this study suggest that this can be sufficiently overcome through the leisure constraint negotiation process. These results strongly support the initial theoretical argument that participation in leisure activities is determined by the negotiation process to overcome the constraints rather than the existence of leisure constraints (Jackson et al., 1993). In line with these results, Kay and Jackson (1991) also claim that participation is not necessarily prevented or not at all hindered by constraints in some cases despite the general assumption that participation in leisure activities can be restricted by constraints in their study on the impact of leisure constraints on leisure participation in England. On the other hand, these conclusions not only have theoretical significance but also practical applications for individuals who wish to lead a continuous leisurely life. Therefore, efforts to overcome the leisure constraints of each participant are desperately required for continuous participation in leisure activities.

Second, the analysis of the effect size between the leisure constraint negotiation sub-factors and leisure participation, there was no significant difference between the effect sizes. This means that the inconsistent results of individual studies on the relationship between the two variables are not explainable by the sub-factors of the leisure constraint negotiation. This result is consistent with the previous study (Han et al., 2018) that observed that there was no significant difference between the sub-factors of the leisure constraint negotiations. Yet, it should be noted that the effect size of each sub-factor of the leisure constraint negotiation was found to be the largest in the leisure activity cost preparation and time management factors. These results suggest that cost and time can act as decisive factors for continued participation in leisure activities. In a similar vein, Shaw et al. (1991) also point out that the social structural constraints including income and occupational status, which are closely related to cost preparation and time management, have impacts on level of participation in leisure activities within the Canadian context. According to the survey data regarding the state of leisure activities of the people, it was observed that “lack of time” and “economic burden” had the greatest influence on dissatisfaction with their leisure lives (Ministry of Culture Sports and Tourism, 2019). Such facts have great implications for institutions that establish leisure policies to support the leisure activities of the people. Therefore, more efforts to establish new and improve existing leisure policies, which consider both leisure time and leisure expenses, should be required.

Third, in the case of gender, men showed a higher effect size than women, and the difference was significant. This means that gender is a variable that regulates the relationship between leisure constraint negotiations and participation in leisure, suggesting that men are more likely to continue leisure activities through the leisure constraint negotiation process than women. These results can be supported by a study of Han et al. (2018), who mentioned that men exert relatively more effort to overcome leisure constraints than women. Jackson and Handerson (1995) also revealed that women are more likely to be constrained in their leisure participation than men in their study on gender-based analysis of leisure constraints within the Canadian context. Considering these facts, it is reasonable to suggest that more ways to improve women’s leisure constraint negotiation strategies are needed. However, considering there is a variation in the number of cases of effect size for gender, it seems that caution in interpreting the study results is necessary. In order to clearly understand whether gender variables regulate the relationship between leisure constraint negotiations and leisure participation, it is necessary to re-verify through additional meta-analysis after more research on male subjects is accumulated.

Fourth, in the case of age, those over 55 years of age showed a higher effect size than those in their 20s, and the difference was significant. This means that age is a variable that controls the relationship between leisure constraint negotiations and leisure participation, which can also be supported by the study of Shaw et al. (1991), and it is possible to explain that older people have a higher relationship between leisure constraint negotiations and leisure participation compared to younger people in their 20s. Perhaps, old age is a time when economic activities are organized, and retired life is reached, or the desire to realize self through leisure activities may be relatively high. In addition, in the case of old age, the physical, psychological, and emotional health conditions are weak, so it can be predicted that there is a strong will to lead a healthy life through leisure activities. This result is supported by Sajin et al. (2016) claim that the old should be encouraged to participate in leisure activities since they can have an emotional involvement and regain their psychological stability, which will lead to the enhancement of their quality of life.

Fifth, one of the main objectives of this study was to determine whether the type of leisure activity regulates the relationship between leisure constraint negotiations and leisure participation. As a result of the analysis, there was no significant difference in effect size according to the type of leisure activity. In other words, the relationship between the two variables was similar in both active and inactive leisure activity participants. This means that the inconsistent results of individual studies on the relationship between leisure constraint negotiations and leisure participation are not explained by the type of leisure activity. These results can be supported by the study of Han et al. (2018), which observed that the desire to overcome leisure constraints did not differ significantly between participants in sports and leisure activities. On the other hand, there was no significant difference between the effect sizes according to the leisure activity type, but the effect size was relatively large in the inactive leisure activity type. This means that inactive leisure activity participants have a closer relationship between leisure constraint negotiations and leisure participation, suggesting that they have a relatively stronger will to overcome leisure constraints than active leisure activity participants. This may be due to the fact that active leisure activities may involve several factors such as travel distance for leisure activities, equipment purchase, facility usage fees, and energy acquisition, while inactive leisure activities are relatively less affected by these factors. However, considering the fact that there are variations in the number of cases of effect size by type of leisure activity, it seems necessary to be careful in interpreting the study results. In order to clearly grasp whether the leisure activity type variable controls the relationship between leisure constraint negotiations and leisure participation, it is necessary to re-verify through additional meta-analysis after more research on inactive leisure activity participants is accumulated.

A final factor analyzed for this study was the effect size on the types of active leisure activities in order to understand in detail the relationship between leisure constraint negotiations and leisure participation according to the types of leisure activities. As a result, skin scuba diving and horseback riding activities were relatively high compared to the other leisure activity choices, and the difference was significant. This means that the type of active leisure activity is a variable that controls the relationship between leisure constraint negotiations and leisure participation, and it suggests that participants engaged in skin scuba diving and horseback riding are relatively more willing to overcome the challenges they face through leisure constraints. However, considering the fact that the number of cases of each effect size is somewhat small and that more diverse types of leisure activities are not included, caution in interpreting the results of this study is considered necessary. In order to more properly explain the relationship between leisure constraint negotiations and leisure participation according to active leisure activities, research on a specific type of leisure activity should be accumulated. In addition, various other types of active leisure activities other than golf, dance, ski, board, skin scuba diving, horseback riding, and bicycle are required to conduct future research.

## Conclusion

The conclusions drawn from the research questions set in this study are as follows. First, leisure constraint negotiations and leisure participation are in a static relationship, and their level is considerable. In other words, efforts to overcome leisure constraints increase participation in leisure activities, which can be supported by findings from previous studies emphasizing the importance of leisure constraint negotiation variables mediating the relationship between leisure constraints and participation in leisure activities (Crawford et al., 1991) and the alleviating role of the negotiation process on participation in leisure activities (Hubbard and Mannell, 2001). Second, gender, age, and type of active leisure activities are major variables that control the relationship between leisure constraint negotiations and leisure participation which have been previously claimed by many scholars [e.g., Jackson and Handerson (1995), Sajin et al. (2016), and Han et al. (2018)].

This study is meaningful in that it comprehensively presents the relationship between the two variables by integrating the quantitative results of individual studies that have observed the relationship between leisure constraint negotiations and participation in leisure. In addition, this study is of academic significance in that it diagnosed the present status of previous studies that observed the relationship between leisure constraint negotiations and leisure participation and suggested the direction of subsequent studies. Finally, the limitations of this study and suggestions for subsequent studies are as follows. First, this study limited the meta-analysis targets to domestic studies (i.e., the Korean context) in order to fully explain the domestic situation on the relationship between leisure constraint negotiations and leisure participation. Even though the results and claims of this study are explained and supplemented by foreign studies along with domestic ones, in subsequent studies, if meta-analysis is performed on foreign studies including domestic studies as well, more meaningful and comprehensive results can be derived through a comparative analysis between domestic and foreign studies. Second, this study drew a comprehensive conclusion on the relationship between leisure constraint negotiations and leisure participation, but this is only possible to explain the relationship between these two variables. However, the factors affecting participation in leisure activities cannot be explained by a single factor alone. Therefore, a follow-up meta-analysis study on various factors related to leisure participation is suggested. Through this, it will be possible to grasp the relative influence of the factors affecting participation in leisure activities.

## Data Availability Statement

The raw data supporting the conclusions of this article will be made available by the authors, without undue reservation.

## Author Contributions

EK and HK: conceptualization, validation, investigation, data curation, and writing-original draft preparation. EK: methodology, formal analysis, and visualization. SP: writing-review and editing. HK: supervision and project administration. All authors contributed to the article and approved the submitted version.

## Conflict of Interest

The authors declare that the research was conducted in the absence of any commercial or financial relationships that could be construed as a potential conflict of interest.

## Publisher’s Note

All claims expressed in this article are solely those of the authors and do not necessarily represent those of their affiliated organizations, or those of the publisher, the editors and the reviewers. Any product that may be evaluated in this article, or claim that may be made by its manufacturer, is not guaranteed or endorsed by the publisher.
